# Depression in Rheumatoid Arthritis and its relation to disease activity

**DOI:** 10.12669/pjms.312.6589

**Published:** 2015

**Authors:** Muhammad Yaser Imran, Elaine Anwer Saira Khan, Nighat Mir Ahmad, Sumaira Farman Raja, Muhammad Ahmed Saeed, Imran Ijaz Haider

**Affiliations:** 1 Dr. Muhammad Yaser Imran, FCPS. Department of Rheumatology, Fatima Memorial Hospital, Lahore, Pakistan; 2 Dr. Saira Elaine Anwer Khan, MRCP. Department of Rheumatology, Fatima Memorial Hospital, Lahore, Pakistan; 3 Prof. Dr. Nighat Mir Ahmad, MD, FACP, FACR. Department of Rheumatology, Fatima Memorial Hospital, Lahore, Pakistan; 4 Dr. Sumaira Farman Raja, MBBS, FRCP, FACP, FACR. Department of Rheumatology, Fatima Memorial Hospital, Lahore, Pakistan; 5 Dr. Muhammad Ahmed Saeed, FCPS medicine, FCPS Rheumatology. Department of Rheumatology, Fatima Memorial Hospital, Lahore, Pakistan; 6 Prof. Dr. Imran Ijaz Haider, MRC Psychiatry. Department of Psychiatry. Fatima Memorial Hospital, Lahore, Pakistan

**Keywords:** Rheumatoid Arthritis, Depression, DAS - 28, BDI scale

## Abstract

**Objectives:**

To determine the level of depression in Rheumatoid Arthritis and its relationship with severity of Rheumatoid Arthritis.

**Methods:**

This cross sectional analytical study was conducted from March 2014 to May 2014.

Total 102 cases of Rheumatoid Arthritis (RA) diagnosed as per ACR (American College of Rheumatology) 1987 criteria were enrolled from Rheumatology Department, Fatima Memorial Hospital. Severity of Rheumatoid Arthritis was assessed by Health Assessment Questionnaire (HAQ) and disease activity score (DAS - 28) while severity of depression was measured by Beck Depression Inventory (BDI) scale II questionnaire. The relationship between depression and disease activity (severity) of Rheumatoid Arthritis was assessed by calculating correlation coefficient between depression, disease activity score (DAS 28) of Rheumatoid Arthritis and health assessment questionnaire (HAQ).

**Results:**

Out of 102 patients, 77(75.5%) were females and 25(24.5%) males. The mean age of patients was 43.5± 11.9 years and the mean duration of disease was 7.8 ± 5.5 years.71.5% of Rheumatoid Arthritis patients were found to have some degree of depression and this was directly related to the severity of disease. Moderate and severe depression were present in 23 (22.5%) and 19(18.6%) patients respectively.

**Conclusion:**

In this study, almost three-fourths of Rheumatoid Arthritis patients were found to have depression. There was a strong association between Rheumatoid Arthritis disease activity and the level of depression. So it is imperative for clinicians treating Rheumatoid Arthritis patients to screen them for co morbid depression and manage it accordingly.

## INTRODUCTION

Rheumatoid Arthritis (RA) is one of the most common forms of chronic polyarticular inflammatory arthritis characterized by persistent synovial inflammation, bony erosions and progressive articular destruction leading to varying degrees of physical disability.^1^ It is characterized by periods of disease flares and remissions.^2^

Rheumatoid Arthritis affects 0.5% to 1% population all over the world. The prevalence of Rheumatoid Arthritis in India (0.75%) is similar to that reported in white population from Manchester U.K (0.8%). In the urban population of Southern Pakistan, Karachi, the prevalence of Rheumatoid Arthritis is reported to be 0.142%, whereas in Northern Pakistan the estimated prevalence is 0.55%.^3^

Co morbid depression has been shown to independently increase work disability in Rheumatoid Arthritis. The loss of recreational and social activities in people with Rheumatoid Arthritis has been shown to significantly increase the risk of depressive symptoms. The coexistence of both conditions adds to the burden of the disease on the health care system as well, with increased physician and general practitioner visits, increased pain complaints and more requests for analgesia. Depression can result in poor adherence to treatment.^4^ Complications associated with depression in Rheumatoid Arthritis patients include an increased risk of work disability, mortality and myocardial infarction.^5^ Beyond the increase in risk for poor health and associated greater health care costs, depression interferes with daily functioning and worsens the quality of life.^6^

Disease activity of Rheumatoid Arthritis is measured by DAS - 28 (disease activity score).^7^ Functional limitations of Rheumatoid Arthritis patients are assessed by HAQ (health assessment questionnaire).^8^ Depression severity is assessed by Beck Depression Inventory scale II questionnaire (BDI-II).^9^

Diagnosing depression in patients with Rheumatoid Arthritis is complicated as there is an overlap of the symptoms of depression and Rheumatoid Arthritis (e.g. fatigue, weight loss, insomnia and lack of appetite) as such the depression frequently goes unrecognized. Use of standardized psychiatric questionnaires like Beck Depression Inventory scale II questionnaire help to assess the severity of depression in these patients.^10^

Rheumatoid Arthritis is commonly associated with depression and as Rheumatology Department of Fatima Memorial Hospital is a specialized referral centre for this disease so this study was conducted here to see the level of depression in patients diagnosed with Rheumatoid Arthritis.

## METHODS

This cross sectional study was performed in Rheumatology Department and Outreach Charity Clinics of Fatima Memorial Hospital over a period of three months from March 2014 to May 2014. Total of 102 patients of Rheumatoid Arthritis from 16 – 65 years of age, of both genders, who were diagnosed according to American College of Rheumatology (ACR) 1987 criteria, were selected by purposive sampling technique. Patients not willing to give written consent or those having malignancies or any other associated diseases were excluded. Signed informed consent was obtained from the patients and confidentiality was maintained. Prior approval by Institutional Review Board of the hospital was taken.

Severity of Rheumatoid Arthritis was assessed by health assessment questionnaire (HAQ) and DAS - 28 (disease activity score). Severity of depression was measured by Beck Depression Inventory scale II questionnaire in which scoring was as follows: 0-13(no depression), 14-19(mild to moderate depression), 20-28 (moderate to severe depression) and 29-63(severe depression). The relationship between depression and disease activity of Rheumatoid Arthritis was assessed. Disease activity was calculated by DAS – 28 score which was as follows: score <2.6: remission, >2.6 and <=3.2: low disease activity, > 3.2 and <=5.1: moderate disease activity, > 5.1: High disease activity. DAS - 28score has four components which are tender joint count, swollen joint count, erythrocyte sedimentation rate (ESR) and visual analogue score (VAS).

Data was analyzed by using SPSS 17 software. Data was stratified on the basis of gender, age, disease duration, Rheumatoid Arthritis (RA) factor status and disease activity. Frequencies were calculated for all categorical variables like sex, social class and RA factor. Correlation coefficient among dependent (depression) and independent (Rheumatoid Arthritis disease activity score i.e. DAS 28) variables was calculated. Mean and standard deviation was calculated for all numerical variables like age, duration of disease, tender and swollen joints count, ESR, VAS and DAS - 28. Chi square test was applied to determine any significance amongst the frequency of depression in patients with high disease activity as compared to the patients with low and moderate disease activity. ANOVA test was applied for the comparison of numerical variables (age, duration of disease, tender joints counts, swollen joints counts, VAS and DAS - 28) between different disease activity groups. A p value of 0.05 or less was taken as significant.

## RESULTS

The mean age of patients was 43.51 + 11.9. Out of 102 patients, 77(75.49%) were females and 25(24.5%) were males. The disease duration was 7.8 ±5.5 years. Three patients had a disease of less than one year while 41 had disease duration of 1 – 5 years and the remaining 58 had Rheumatoid Arthritis for more than five years. The mean and standard deviation of different variables are shown in Table-I.

**Table-I:** Patient’s demographic and RA specific variables.

Variables	Mean±Std.Deviation
Age (Years)	43.51 ± 11.90
Disease duration (years)	7.80 ± 5.54
Tender Joint counts (TJC)	4.54 ± 4.35
Swollen Joint counts (SJC)	2.60 ± 3.18
ESR (Erythrocyte sedimentation rate)	39.76 ± 24.84
DAS 28	4.37 + 1.38
VAS	37.28 ± 25.76
Social Status	33260.00 ± 40428.67
HAQ	2.86 ± 4.371
RA factor	100%

TJC (Tender joint count), SJC (Swollen joint count),

ESR (Erythrocyte Sedimentation Rate),

DAS 28(Disease activity score),

VAS (Visual analogue scale),

HAQ (Health Assessment Questionnaire),

RA (Rheumatoid Arthritis)

The results of severity of depression as measured by Beck Depression Inventory (BDI) scale II questionnaire are as follows: Out of 102 patients, 31(30.39%) patients had mild depression, 23(22.54%) had moderate depression and 19(18.62%) patients had severe depression while 29(28.43%) patients had no depression.

The disease activity of Rheumatoid Arthritis as measured by DAS– 28 score was as follows: 33(32.35%) patients had low disease activity, 34(33.33%) patients had moderate disease activity and 35(34.31%) patients had high disease activity.

Health Assessment Questionnaire (HAQ) was found to be positively correlated with (severity of depression) BDI score (r = 0.48, p value = 0.001), further strengthening our understanding that active Rheumatoid Arthritis not only leads to disability but also can contribute to depression.

**Table-II:** Comparison between different rheumatoid arthritis disease activity subgroups with respect to severity of depression.

Severity of depression	DAS 28 score		
	Low N = 33 (32.35%)	Moderate N = 34 (33.33%)	High N = 35 (34.31%)
No	19 (57.6%)	5 (14.7%)	5 (14.3%)
Mild	6 (18.2%)	16 (47.1%)	9 (25.7%)
Moderate	5 (15.2%)	9 (26.5%)	9 (25.7%)
Severe	3 (9.1%)	4 (11.8%)	12 (34.3%)

Pearson chi square = 27.81, P value = < 0.001,

This p value is calculated by Pearson chi square test.

## DISCUSSION

This study is unique because it not only shows the level of depression in patients suffering from Rheumatoid Arthritis but also establishes its association with disease activity and disability. To date two studies on depression have been carried out in Pakistan at a rheumatology clinic. In one study only gender was associated with depression.^11^ Another study negated this association and found disease activity, duration and disability to be directly related to depression.^12^

Literature search showed major depressive disorder to be common in patients with Rheumatoid Arthritis, with a prevalence of 13–42%, at least two to four-times that in the general population.^13^ In our study about 70% patients were reported depressed. This level was similar to that established in previous studies in this region. In another study, the prevalence of depression in Rheumatoid Arthritis was 65% that was close to our results.^14^ The reason for this high frequency of depression is almost twice that seen in developed countries, is multi-factorial. In our study out of 102 patients, 77 (75.5%) were females. This is in line with most of the studies done using purposing sampling as Rheumatoid Arthritis is a female predominant disease.^15^

**Fig.1:**
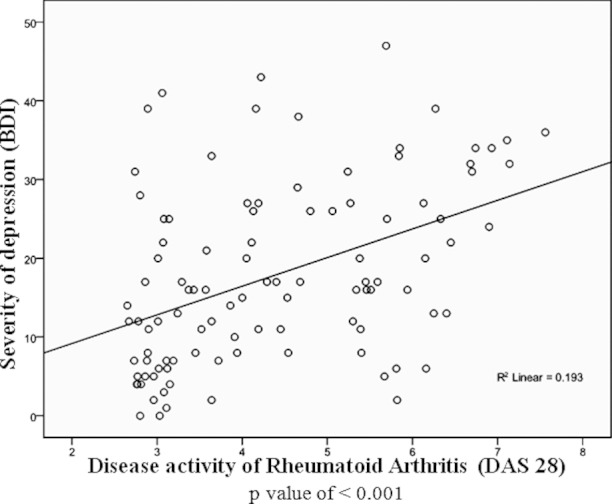
Correlation between severity of depression and disease activity score of rheumatoid arthritis.

In our study the relationship between severity of depression and activity of Rheumatoid Arthritis was linear with a significant p value of <0.0001 as shown by Fig.1. Majority (67.6%) of our Rheumatoid Arthritis patients had active disease and would have high inflammatory cytokines like TNF and IL-6 which is also contributing to depression. Majority (67.6%) had DAS - 28 score ≥3.2.^16^ This may be one of the reasons why in our study, about 2/3^rd^ (71%) of patients had depressive symptoms.

**Fig.2:**
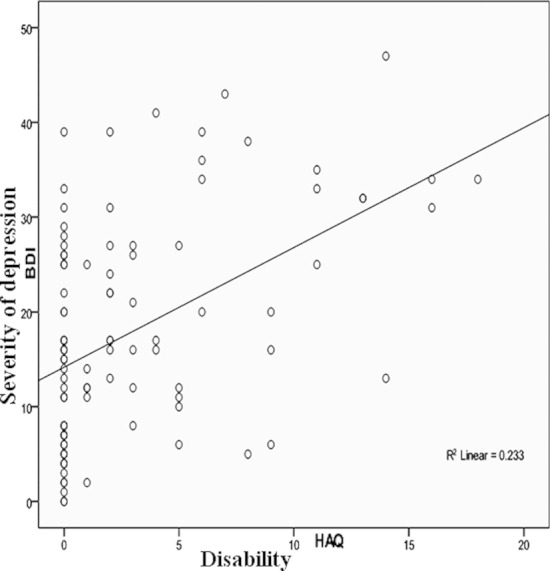
Correlation between severity of depression and health assessment questionnaire.

There is no doubt that limited function, as measured by the Health Assessment Questionnaire, is a strong predictor of depression in patients with Rheumatoid Arthritis.^17^ In our study HAQ was found to be positively correlated with(severity of depression) BDI II scale (r = 0.48, p value = 0.001) shown by Fig.2, further compounding the burden of depression. Mean HAQ score of 2.86 ± 4.371 is well above the previously reported mean HAQ score of 0.71 in a population-based cohort of patients with Rheumatoid Arthritis.^18^

Assessing depressive symptoms in patients with Rheumatoid Arthritis can be difficult. It is well understood that somatic symptoms of depression (e.g., fatigue or decreased energy) overlap with symptoms of Rheumatoid Arthritis. Consequently, there is a risk that depression in Rheumatoid Arthritis may be overestimated. Different studies have used different instruments/scales for assessing depression. So it is difficult to compare results. In our study we used Beck Depression Inventory (BDI) scale II questionnaire to see the level of depression. Beck Depression Inventory (BDI) II scale has high specificity 78.4% vs 72.7% sensitivity for detecting depression. Nevertheless, it has been suggested that the Beck Depression Inventory (BDI) scale overestimates incidence of depression in pain patients. When using Beck Depression Inventory (BDI) scale for screening purposes with a pain clinic population, a cut-off of 22 or above has been suggested.^19^ If this cut-off is applied then it would lead to a reduction of 31% in the reported depression level, leading to a depression level of 40%. This level is similar to that reported in developed countries.^20^

As our study has been conducted in a rheumatology division of a tertiary care hospital where mostly patients seen might have been referred with moderate to severe disease, there is a chance of overestimating the prevalence of depression as compared to Rheumatoid Arthritis patients in community. There is a possibility that most patients having severe, aggressive or refractory disease are being referred leading to higher proportion of depression in our out patient population. This presumption needs further studies to address this factor in Primary care or Community settings.

In our study, majority of our patients belonged to lower socio economic status which may have influenced the number of patients with depression. There are significant number of studies done linking socio-economic status with depression and Rheumatoid Arthritis.^21^

Remission in Rheumatoid Arthritis leading to improved function will keep reducing the burden of depression in Rheumatoid Arthritis. So we recommend that clinicians taking care of Rheumatoid Arthritis patients should treat them aggressively as per” Treat to Target” approach suggested by EULAR(European League Against Rheumatism) and ACR(American College of Rheumatology) guidelines for treatment of Rheumatoid Arthritis.^22^,^23^ Remission or low disease activity should be goal for treating physician.

## CONCLUSION

In this study, almost three-fourth of Rheumatoid Arthritis patients were found to have depression. There was a strong association between Rheumatoid Arthritis disease activity and the level of depression. Remission of Rheumatoid Arthritis or low disease activity should be a goal for treating physicians. So it is imperative for clinicians treating Rheumatoid Arthritis patients to screen them for co morbid depression and manage it accordingly.
